# Absence of seroreversion in 80 HAART-treated HIV-1 seropositive patients with at least five-years undetectable plasma HIV-1 viral load

**DOI:** 10.1186/1742-6405-3-3

**Published:** 2006-02-16

**Authors:** Marion Cornelissen, Suzanne Jurriaans, Jan M Prins, Margreet Bakker, Antoinette C van der Kuyl

**Affiliations:** 1 Department of Human Retrovirology, Academic Medical Centre, University of Amsterdam, Amsterdam, The Netherlands; 2 Department of Internal Medicine, Division of Infectious Diseases, Tropical Medicine and AIDS, Academic Medical Centre, University of Amsterdam, Amsterdam, The Netherlands

## Abstract

Partial or complete seroreversion for HIV-1, or incomplete antibody evolution are relatively rare events that have so far only been described in patients treated with HAART early after virus infection. Whether seroreversion is seen in patients treated effectively with HAART years after their acute infection has not been investigated so far. Therefore we have investigated anti-HIV antibody levels in 80 patients treated with HAART during chronic HIV-1 infection, who had an undetectable HIV-1 plasma viral load for at least five years. In none of the patients we observed seroreversion, and there was also no significant decrease or increase in antibody levels in this group of patients. So, successful HAART treatment during chronic HIV-1 infection does not induce seroreversion.

## Findings

Seroreversion, defined as a quantitative decrease in specific antibody levels so that they measure below the cutoff of an assay, can be partial, resulting in the loss of response against one or a few antigens, or complete, with loss of total antibody reactivity. In HCV infection, seroreversion, which is found in 16–23% of the patients, has been associated with virus clearance, but it can also be observed in chronic HCV infection [1,2]. HCV seroreversion can occur spontaneously, in association with immune-suppression, or after antiviral treatment [3]. HCV seroreversion is often transient, suggesting that antibody levels fluctuate around the cutoff of the assay [2]. In HIV infection, both partial and complete seroreversions are rare. Apart from being documented in non-infected babies of seropositive mothers due to loss of maternal antibodies [4-6], seroreversion was seen in late-stage AIDS patients [7], in neonates treated very early with HAART [8], and in patients treated with antiviral therapy during acute infection [9-11]. In these patients, some seroreversions were partial (incomplete evolution of the western blot pattern), and some were complete (negative in an HIV-1/2 ELISA). Transient seroreversion has been reported in a single HIV-1 infected patient [12]. The clinical significance of HIV seroreversion is unclear [13,14], as is the frequency of seroreversion in chronic HIV-1 infection. It could be assumed that a loss of antibody response is related to a loss in antigenic stimuli, suggesting that seroreversion indicates an absence of viral replication. Indeed, HCV seroreversion is accompanied in many cases by the absence of viral RNA as detected by PCR, although in many other viral infections, clearance of the virus does not induce loss of antibodies. Over the last decade, treatment of HIV infected patients with antiviral drugs often results in long-term undetectable HIV viral load in plasma. Viremia in untreated patients probably results from both active replication as well as release of HIV-1 RNA from stable reservoirs, e.g. memory T-cells, while in treated patients there is only low-level viral RNA release from these reservoirs (reviewed by [15]). Currently, there is no evidence suggesting that clearance of HIV is achievable, and attempts at viral eradication have failed so far [15]. Even in treated patients with long-term undetectable plasma viral load, transient elevations of the viral load into the detectable range of the assay, so-called blips, are common. So, it is likely that even HIV-1 infected patients with optimal treatment response experience low-level HIV-1 activity, which would preclude seroreversion. However, it cannot be excluded that patients with no replication of HIV do exist in this patient group.

To examine the effect of long-term undetectable plasma HIV-1 levels upon the serological response, we have analysed the HIV-1/HIV-2 antibody levels in 80 patients treated with HAART resulting in at least a five years undetectable plasma viral load (<50 copies/ml) without blips > 400 copies/ml. Forty-four patients (55%) had an undetectable HIV-1 load for more than 7 years. Fifty patients showed one or two blips of ≤ 400 copies/ml during these years, thirty patients experienced no blips. Presumably, patients with blips have a higher mean residual viremia than patients without blips [16]. Plasma HIV-1 RNA was measured using the VERSANT HIV-1 RNA 3.0 assay (bDNA) (Bayer Diagnostics Division, Tarrytown, NY, USA), which has a detection level of 50 copies/ml. Plasma HIV-1 RNA levels were determined every four months for at least 8 years (since the start of HAART) in this patient group. The HIV-1 antibody ratio in serum was measured with the IMx System (IMx System HIV-1/HIV-2 III Plus, ABBOTT Laboratories, Abbott Park, Il, USA) in the samples prior to the start of HAART, and five years later. The reagents used in the HIV IMx assay include recombinant HIV transmembrane glycoproteins (expressed in *E. coli *and *B. megaterium*), HIV-1 p24 (expressed in *E. coli*), and synthetic peptides from HIV-1 gp41 and HIV-2 gp36. This assay is available as a qualitative test, but is in fact a kinetic assay whereby increasing amounts of a product are formed with time, and are monitored at multiple points (personal communication, ABBOTT Laboratories), suggesting it can be used as a semi-quantitative analysis.

All patients tested HIV seropositive at baseline and after at least five years of undetectable plasma HIV-1 load, irrespective of the occurrence of blips. Table 1 summarizes the IMx antibody measurements for all patients. No significant difference in antibody levels was seen after five years of HAART (average IMx ratio = 34.87 at the start of HAART, average IMx ratio = 34.93 after five years, p = 0.95). Separating the groups with and without blips did not reveal either any significant difference in IMx ratio after five years of treatment (Table 1). Although no patient showed evidence for seroreversion, there were 37 (46%) patients in total with a decrease in IMx ratio after five years of HAART, without a significant difference between the blip group (19 out of 50 patients (38%), average decrease in IMx ratio of 8.58) and non-blip group (18 out of 30 patients (60%), average decrease in IMx ratio of 6.36, p-value = 0.056 (chi-square test) for patients numbers with decreasing antibody levels in each group).

**Table 1:** HIV-1 antibody measurements in HIV-1 infected patients with ≥ five-years undetectable viral load

Group	N =	Average IMx ratio^1 ^at start of HAART, ± st dev (range)	Average IMx ratio ≥ 5 years undetectable viral load, ± st dev (range)	P-value^2^
Patients without blips	30	37.48 ± 9.93 (16.83–52.33)	36.02 ± 7.79 (17.25–45.46)	P = 0.95
Patients with 1–2 blips	50	33.30 ± 9.27 (13.08–53.39)	34.27 ± 7.56 (15.22–47.59)	P = 0.29
All patients	80	34.87 ± 9.68	34.93 ± 7.65	P = 0.46

^1 ^Antibody levels are calculated as ratios of the sample rate divided by the cutoff (= negative control). A sample is considered non-reactive if the ratio <1, and reactive when the ratio ≥ 1. The assay cutoffs are determined at each run, and vary slightly per assay and over time.

^2 ^Two-tailed student's t-test.

From this study it is clear that seroreversion is not common in HIV infection, not even after the achievement of prolonged low plasma HIV-1 RNA levels, as it was not seen in our group of 80 patients with long-term undetectable HIV-1 load. Earlier observations on HIV seroreversion suggested that seroreversion could occur when HAART is given during acute infection. Our study suggests that HAART given during chronic infection does not induce seroreversion. As seroreversion has been associated with viral clearance, its absence during successful HAART treatment possibly reflects the low level HIV-1 replication in these patients.

## Competing interests

The author(s) declare that they have no competing interests.

## List of abbreviations

HCV: hepatitis C virus. HIV: human immunodeficiency virus. AIDS: acquired immunodeficiency syndrome. HAART: highly active antiretroviral therapy. ELISA: enzyme-linked immunosorbent assay.

## Authors' contributions

MC conceived of the study, and participated in its design and coordination, SJ carried out the immunoassays and participated in the design of the study, JMP is the treating physician who collected the samples, MB selected the patients for the study, and ACvdK participated in the design of the study, carried out the statistical analyses and drafted the manuscript. All authors read and approved the final manuscript.

## References

[B1] Lanotte P, Dubois F, Le Pogam S, Guerois C, Fimbel B, Bacq Y, Gruel Y, Goudeau A, Barin F (1998). The kinetics of antibodies against hepatitis C virus may predict viral clearance in exposed hemophiliacs. J Infect Dis.

[B2] Marinho RT, Pinto RM, Santos ML, de Moura MC (2004). Lymphocyte T helper-specific reactivity in sustained responders to interferon and ribavirin with negativation (seroreversion) of anti-hepatitis C virus. Liver Int.

[B3] Lefrere JJ, Guiramand S, Lefrere F, Mariotti M, Aumont P, Lerable J, Petit JC, Girot R, Morand-Joubert L (1997). Full or partial seroreversion in patients infected by hepatitis C virus. J Infect Dis.

[B4] Lepage P, P. VP, Simonon A, Msellati P, Hitimana DG, Dabis F (1992). Transient seroreversion in children born to human immunodeficiency virus 1-infected mothers. Pediatr Infect Dis J.

[B5] Chantry CJ, Cooper ER, Pelton SI, Zorilla C, Hillyer GV, Diaz C (1995). Seroreversion in human immunodeficiency virus-exposed but uninfected infants. Pediatr Infect Dis J.

[B6] Bakshi SS, Tetali S, Abrams EJ, Paul MO, Pahwa SG (1995). Repeatedly positive human immunodeficiency virus type 1 DNA polymerase chain reaction in human immunodeficiency virus-exposed seroreverting infants. Pediatr Infect Dis J.

[B7] Gutierrez M, Soriano V, Bravo R, Vallejo A, Gonzalez-Lahoz J (1994). Seroreversion in patients with end-stage HIV infection. Vox Sang.

[B8] Hainaut M, Peltier CA, Goetghebuer T, Van der LD, Marissens D, Zissis G, Levy J (2005). Seroreversion in children infected with HIV type 1 who are treated in the first months of life is not a rare event. Clin Infect Dis.

[B9] Lafeuillade A, Poggi C, Tamalet C, Profizi N, Tourres C, Costes O (1997). Effects of a combination of zidovudine, didanosine, and lamivudine on primary human immunodeficiency virus type 1 infection. J Infect Dis.

[B10] Jurriaans S, Sankatsing SU, Prins JM, Schuitemaker H, Lange J, van der Kuyl AC, Cornelissen M (2004). HIV-1 seroreversion in an HIV-1-seropositive patient treated during acute infection with highly active antiretroviral therapy and mycophenolate mofetil. AIDS.

[B11] Kassutto S, Johnston MN, Rosenberg ES (2005). Incomplete HIV type 1 antibody evolution and seroreversion in acutely infected individuals treated with early antiretroviral therapy. Clin Infect Dis.

[B12] Zaaijer HL, Bloemer MH, Lelie PN (1997). Temporary seronegativity in a human immunodeficiency virus type 1-infected man. J Med Virol.

[B13] Connick E (2005). Incomplete antibody evolution and seroreversion after treatment of primary HIV type 1 infection: what is the clinical significance?. Clin Infect Dis.

[B14] Crabb C (2005). Seroreversion in patients receiving HAART during acute infection 2. AIDS.

[B15] Siliciano RF (2005). Scientific rationale for antiretroviral therapy in 2005: viral reservoirs and resistance evolution. Top HIV Med.

[B16] Di Mascio M, Markowitz M, Louie M, Hurley A, Hogan C, Simon V, Follmann D, Ho DD, Perelson AS (2004). Dynamics of intermittent viremia during highly active antiretroviral therapy in patients who initiate therapy during chronic versus acute and early human immunodeficiency virus type 1 infection. J Virol.

